# Glucuronolactone Restores the Intestinal Barrier and Redox Balance Partly Through the Nrf2/Akt/FOXO1 Pathway to Alleviate Weaning Stress-Induced Intestinal Dysfunction in Piglets

**DOI:** 10.3390/antiox14030352

**Published:** 2025-03-18

**Authors:** Beibei Zhang, Min Tian, Yueqin Qiu, Jing Wu, Chenbin Cui, Shilong Liu, Jing Hou, Chaoyang Tian, Li Wang, Kaiguo Gao, Zongyong Jiang, Xuefen Yang

**Affiliations:** 1 College of Animal Science, South China Agricultural University, Guangzhou 510642, China; 2 Institute of Animal Science, Guangdong Academy of Agricultural Sciences, Guangzhou 510640, China; 3 State Key Laboratory of Swine and Poultry Breeding Industry, Guangzhou 510640, China; 4 Key Laboratory of Animal Nutrition and Feed Science in South China, Ministry of Agriculture and Rural Affairs, Guangzhou 510640, China; 5 Guangdong Provincial Key Laboratory of Animal Breeding and Nutrition, Guangzhou 510640, China

**Keywords:** weaned piglet, glucuronolactone, oxidative stress, growth performance, intestinal health

## Abstract

(1) Background: Glucuronolactone (GLU) is a glucose metabolite with antioxidant activity. At present, the exact role of it in regulating the intestinal health of piglets under weaning stress is not clear. The purpose of this study is to investigate the effects of GLU on the growth performance and intestinal health of piglets under weaning stress and to explore potential mechanisms. (2) Methods: Twenty-four weaned piglets were randomly assigned into two groups, with one group receiving a basal diet and the other group receiving an experimental diet supplemented with 200 mg/kg of GLU. (3) Results: GLU increased the ADG, ADFI, and final body weight of piglets, while reducing the diarrhea rate. Mechanistically, GLU alleviates weaning stress-induced intestinal oxidative stress and inflammatory responses in piglets partly through activating the Nrf2-Akt signaling pathway to suppress the transcriptional activity of FOXO1, while also inhibiting the activation of the TLR4-MAPK signaling pathway to reduce the secretion of pro-inflammatory cytokines. Moreover, GLU increased the relative abundance of *Lactobacillus reuteri* in the ileum of piglets and improved the composition of the gut microbiota. (4) Conclusions: GLU reduced inflammation and oxidative stress through the Nrf2/Akt/FOXO1 signaling pathway and improved intestinal health, resulting in improved growth performance of the piglets.

## 1. Introduction

The gastrointestinal tract is not only the main place for the digestion and absorption of nutrients but also an important immune organ, which is vital for resisting ingested harmful substances to ensure health [1]. However, the intestines of early weaned piglets are not fully developed, which poses a significant health risk to post-weaning piglets [2]. Many factors, including malnutrition, oxidative stress, inflammation, and bacterial infection, are detrimental to the integrity of the intestinal barrier in early-weaned piglets [3,4]. The imbalance of redox reactions is an important intrinsic factor leading to an impairment of the intestinal function in piglets [5]. Oxidative stress is a result of the excessive accumulation of reactive oxygen species (ROS) in cells and is a common feature of many chronic and acute intestinal diseases [6]. The excessive accumulation of ROS in cells disrupts cellular functions and induces apoptosis, which in turn leads to atrophy of the intestinal mucosa and diminishes the intestinal ability to digest and absorb nutrients [7,8]. As a result, the intestinal resistance to harmful substances and pathogenic bacteria is reduced. Therefore, adopting nutritional strategies to reduce intestinal oxidative stress in piglets is key to helping them successfully pass through the weaning period.

In order to alleviate oxidative stress, some natural antioxidants have been gradually discovered, such as resveratrol [9,10], baicalin [11,12,13], and glucuronolactone (GLU) [14,15]. This study focused on the effects of GLU in piglets. GLU is an important component of connective tissue and collagen in animals and is mainly produced via glucose metabolism in the liver, with the chemical formula C_6_H_8_O_6_ [16,17]. GLU is converted to glucuronic acid when it enters the body through oral intake [17]. Studies have found that glucuronic acid is involved in glucose metabolism and is the direct precursor of ascorbic acid formation in organisms [16]. Both -CHO and -COOH exist in the molecule of glucuronic acid, which can react with toxic metabolites, such as -OH, -CH_2_OH, and -SH, in the body to form non-toxic or low-toxic compounds excreted in urine and bile [18]. In addition, glucuronic acid is a powerful antioxidant that scavenges free radicals and reduces oxidative damage [19]. It can also reduce inflammatory responses by downregulating interleukin-6 (IL-6) and nuclear factor-kappa B (NF-κB) [15]. Studies have found that GLU has hepatic-protective and antioxidant activities and antiapoptotic effects in Ochratoxin A (OTA)-treated cultured hepatocytes of chickens [14]. Therefore, GLU is expected to be used as a green feed additive to improve the health of weaned piglets. Currently, the application effect of GLU in pig production is still unclear. This study aimed to evaluate the effects of GLU on the growth performance and intestinal health of weaned piglets, while exploring its potential regulatory mechanisms. This study will provide a theoretical basis for optimizing the nutritional plans for piglets in the future.

## 2. Materials and Methods

### 2.1. Animal Experiment

#### 2.1.1. Ethical Statement

The experiment was conducted from May to June 2024 in the piglet housing facility at the Institute of Animal Science, Guangdong Academy of Agricultural Sciences. All animal protocols in this study were approved by the Animal Care and Use Committee of the Guangdong Academy of Agricultural Sciences (no. 2024011) and under the guidance of the Animal Management Regulations of China.

#### 2.1.2. Animals and Experimental Design

A total of twenty-four piglets aged 21 days (6.33 ± 0.13 kg) were randomly divided into two groups based on their gender and initial weight, with six replicates per group and 2 piglets per litter (one male and one female). The piglets in the control group (CON) were provided with a basal diet, whereas those in the experimental group (GLU) received the same basal diet supplemented with 200 mg/kg of GLU (containing ≥ 98%, supplied by Zhucheng Haotian Pharmaceutical Co., Ltd., Zhucheng, Shandong, China). To determine the optimal dosage of GLU in vivo, we conducted a gradient experiment prior to this study. Results showed that GLU supplementation within the range of 100–500 mg/kg significantly improved the growth performance of piglets. Therefore, in this experiment, we selected a moderate dose of 200 mg/kg GLU for an in-depth mechanistic investigation. The nutritional level of the basal diet met the recommended values for 7–11 kg pigs in the National Research Council (2012) [20], and the ingredients and nutrient levels of the basal diets are shown in Table 1. Each pen was equipped with a 1.8 × 2.5 m fully-slatted leaky floor measuring, a single-sided feeder, and two stainless steel nipple drinkers. The room temperature was set at 28 ± 1 °C during the first week after weaning. Afterwards, the room temperature was lowered by 0.5 °C per day from the second week until a constant 25 °C was reached. All piglets were fed the experimental diets directly after weaning and were provided ad libitum access to feed and water during the experiment.

#### 2.1.3. Data and Sample Collection

On the morning of the first and 22nd day of the experiment, the initial and final weights of piglets were measured after a 12 h fasting period. During the experiment, feed consumption was recorded for each pen of piglets. Based on the above data, the piglets’ average daily feed intake (ADFI), average daily gain (ADG), and feed conversion ratio (G/F) were calculated. Furthermore, the diarrhea rate per pen of piglets was determined with reference to a previous study [21]. At the end of the trial, blood samples were collected from the anterior vena cava of piglets using heparin-sodium-containing vacuum tubes. Then, these blood samples were centrifuged for 15 min at 3000 rpm to obtain plasma. Finally, all piglets were euthanized through the injection of pentobarbital sodium (body weight, 10 mg/kg). According to the method used in previous studies [22], intestinal samples were collected from the middle duodenum (10 cm distal to the pylorus), the middle jejunum (50 cm distal to the pylorus), and the distal ileum (10 cm proximal to the ileocecal junction) of piglets. Among them, the samples designated for intestinal morphology analysis were fixed in a 4% paraformaldehyde solution. After cleaning the intestinal tissue with physiological saline (0.9% NaCl), intestinal mucosal samples were collected with glass slides. Subsequently, these mucosa samples were stored in a −80 °C freezer until analysis.

#### 2.1.4. Serum Biochemical Indicators

The serum concentrations of total protein (TP), albumin (ALB), alanine aminotransferase (ALT), aspartate aminotransferase (AST), total bilirubin (TBili), alkaline phosphatase (ALP), total antioxidant capacity (T-AOC), malondialdehyde (MDA), total superoxide dismutase (T-SOD), and catalase (CAT) were determined with the kits procured from Nanjing Jiancheng Bioengineering Research Institute (Nanjing, Jiangsu, China). Moreover, the concentrations of diamine oxidase (DAO), D-lactate, interleukin-1β (IL-1β), interleukin-10 (IL-10), interleukin-22 (IL-22), transforming growth factor-beta (TGF-β), tumor necrosis factor-alpha (TNF-α), interleukin-6 (IL-6), secretory immunoglobulin A (sIgA), immunoglobulin A (IgA), immunoglobulin G (IgG), and immunoglobulin M (IgM) in serum were detected using the ELISA kits supplied by Jiangsu Meimian Industrial Co., Ltd. (Yancheng, Jiangsu, China). The information of the kits used in our study are shown in Table S1.

#### 2.1.5. Measurement of Intestinal Transmembrane Electrical Resistance

With reference to previous studies [8], 6 cm segments of the proximal jejunum were obtained, then washed in Ringer’s solution, and subsequently mounted onto an EasyMount Ussing chamber system (model VCC MC6, San Diego, CA, USA) for assessment [23]. After a 2 h equilibration period, transepithelial electrical resistance (TEER), expressed in ohms per square centimeter (Ω·cm^2^), was measured every 15 min over three consecutive intervals, and the results were averaged.

#### 2.1.6. Intestinal Morphology Analysis

The tissues of the duodenum, jejunum, and ileum intended for morphological analysis were fixed in a 4% paraformaldehyde solution for a minimum of 48 h. Following standard paraffin embedding techniques [24], the intestinal tissues were processed by embedding them in paraffin and sectioning using a microtome (Leica RM2235, Wetzlar, Germany). The sections were then stained with hematoxylin and eosin for a histopathological analysis. Subsequently, the stained tissue sections were imaged using a digital microscope slide scanner (Pannoramic 250, 3D HISTECH, Budapest, Hungary). Six slices were analyzed in each group, and three fields of view were randomly selected from each slice. From each field, three well-oriented crypt–villi units were randomly selected. Using Image J Pro Plus 6.0 software, the villus height and crypt depth were measured. Subsequently, the ratio of the villi height to crypt depth was calculated.

#### 2.1.7. Intestinal Immunohistochemical Analysis

The intestinal tissue slices used for immunohistochemical analysis were processed based on previous studies [25]. Firstly, the intestinal tissue slices were fixed in 10% neutral buffered formalin. Then, these slices were incubated in xylene for dewaxing and dehydrated with different concentrations of ethanol, and the antigen was recovered in citrate buffer solution. Next, the slices were stained using a streptavidin–biotin–peroxidase complex (ABC) method [26]. The details of the antibodies used in this study are shown in Table S1.

#### 2.1.8. Total RNA Extraction and Real-Time Quantitative PCR

The jejunum and ileum mucosa (100 mg) were homogenized in 1 mL of Trizol reagent (Invitrogen, Carlsbad, CA, USA) using an automatic low-temperature homogenizer (Jingxin, Shanghai, China). After standing for 5 min, the mixture was centrifuged at 12,000 rpm for 15 min at 4 °C to obtain the supernatant. Total RNA was then extracted from the supernatant using an RNA Purification Kit (EZB-RN001, EZBioscience, Roseville, MN, USA). The purity and concentration of the total RNA were measured using a NanoDrop ND-1000 spectrophotometer (NanoDrop Technologies, Wilmington, DE, USA). Subsequently, the first-strand cDNA was synthesized using a Takara Reverse Transcription Kit (Takara, Tokyo, Japan) following the manufacturer’s protocol. The relative expression of target genes was quantitatively analyzed using a Bio-Rad C1000 Touch Thermal Cycler (Bio-Rad Laboratories, Richmond, CA, USA) with a SYBR Green chemistry kit (Bio-Rad Laboratories, Richmond, CA, USA). The reaction mixture consisted of SYBR Green chemistry (10 μL), double-distilled water (8.6 μL), PCR primers (10 μM, 0.2 μL each), and cDNA template (1 μL). The PCR procedure included the following steps: pre-denaturation at 95 °C for 30 s, denaturation at 95 °C for 5 s, annealing at 60 °C, and extension for 30 s, repeated for 40 cycles.

The primers were designed using Primer 5.0 software (Premier Biosoft International, PREMIER Biosoft International, San Francisco, CA, USA) and synthesized by Shanghai Sangon Biotech Co., Ltd. (Shanghai, China). Melting curve analysis was applied to confirm the efficiency and specificity of the qPCR primers [27]. The information of the primers is shown in Table 2. Data normalization was achieved against the internal control gene β-actin, and the relative expression of target genes was analyzed according to the 2^−ΔΔCt^ method, where ΔΔCT = (CT_Target_ − CT_β-actin_) Treatment − (Average CT_Target_ − Average CT_β-actin_) Control.

#### 2.1.9. Chemical Analysis of Intestinal Mucosa

The intestinal mucosa (100 mg) was homogenized in 0.9 mL of 1× PBS using an automatic low-temperature homogenizer (Jingxin, Shanghai, China). The resulting mixture was then centrifuged at 3000 rpm for 10 min at 4 °C to obtain the supernatant. Next, the supernatant was separated using a pipette for subsequent analysis. The concentrations of T-AOC, T-SOD, CAT, MDA, IL-6, IL-22, TNF-α, and sIgA in the mucosa of the jejunum and ileum were detected using the ELISA kits purchased from Jiangsu Meimian Industrial Co., Ltd. (Yancheng, Jiangsu, China). The information of kits used in this study are shown in Table S1.

#### 2.1.10. Microbiota 16S rRNA Sequencing and Analysis

The genomic DNA of microbes from ileal and colonic contents was extracted using the Cetyltrimethylammonium Bromide (CTAB) method, and its quality was evaluated through agarose gel electrophoresis. The V3–V4 region of the 16S rRNA gene was then amplified using universal primers: forward primer 341F (5′-ACTCCTACGGGAGGCAGCA-3′) and reverse primer 806R (5′-TCGGACTACHVGGGTWTCTAAT-3′) [24]. Sequencing of the 16S rRNA gene amplicons was performed on the Thermo Fisher Ion S5TM XL platform (Novogene, Beijing, China) based on amplicon sequence variants (ASVs). To ensure data quality, low-quality bases in the raw reads were filtered out using Cutadapt software (version 1.9.1), and reads were demultiplexed according to barcode sequences. Operational taxonomic units (OTUs) were clustered using Uparse software (version 7.0.1001), with OTUs defined at a 97% sequence identity threshold using USEARCH software version v.10. Alpha diversity was assessed using the Simpson, Shannon, and Chao 1 indices, while beta diversity was analyzed through principal coordinate analysis (PCoA).

#### 2.1.11. Network Pharmacological Analysis

Potential targets of GLU were identified using the Traditional Chinese Medicine Systems Pharmacology Database and Analysis Platform (TCMSP). To obtain these targets, several databases were utilized, including the Therapeutic Target Database (https://db.idrblab.net/ttd/, accessed on 12 June 2024), OMIM (https://omim.org/, accessed on 26 June 2024), Comparative Toxicogenomics Database (https://ctdbase.org/, accessed on 21 June 2024), GeneCards (https://www.genecards.org/, accessed on 2 July 2024), and DisGeNET (https://www.disgenet.org/, accessed on 11 July 2024). Gene information related to inflammation and oxidative stress was also retrieved from these databases. Using bioinformatics technology, a Venn diagram was created, and the overlapping genes were exported to an Excel file as potential targets [28].

Subsequently, the target information for GLU related to oxidative stress and inflammation was imported into the STRING database (https://string-db.org/, accessed on 2 July 2024) to generate protein–protein interaction (PPI) data, and a TSV file was downloaded [29]. This file was then visualized using Cytoscape 3.10 software (Case Viewer 2.3, 3D HISTECH, Budapest, Hungary) to construct the protein interaction network. Topological assessments were conducted to identify central targets. Finally, pathway enrichment analysis of the overlapping targets was performed using Kyoto Encyclopedia of Genes and Genomes (KEGG) bioinformatics techniques.

#### 2.1.12. Western Blot Analysis of Intestinal

The total protein was extracted from the intestinal mucosa using a mixture of RIPA lysis buffer (Beyotime, Shanghai, China) supplemented with 1% PMSF protease inhibitor and 1% phosphatase inhibitors (Biosharp Life Sciences, Hefei, Anhui, China). The protein concentration in the supernatant was measured using a BCA Protein Assay Kit (Beyotime, Shanghai, China) following centrifugation. Then, a certain amount of total protein, RIPA buffer, and protein loading buffer were mixed to prepare a sample with an equal protein concentration and denatured at 100 °C. Next, the samples containing 25 μg of total protein was separated on 10% SDS-PAGE gels (Beyotime, Shanghai, China) and electrically transferred to polyvinylidene difluoride membranes (Millipore, Bedford, MA, USA). After being blocked with 5% non-fat milk, these membranes were immersed sequentially in the primary antibody (1:1000 dilution) and the secondary antibody (1:5000 dilution). In this study, all Western blot analyses included four replicate samples per group. The information regarding the primary and secondary antibodies utilized in this study can be found in Table S2. Finally, the band signals of these membranes were captured using the Chemiluminescence Image Analysis System (Thermo Fisher Scientific, Wilmington, DE, USA) and an ECL chemiluminescence solution (Beyotime, Shanghai, China). The gray values of these bands were analyzed using Image J Software (ImagePro Plus 6.0, Media Cybernetics, Inc., Rockville, MD, USA), and the relative protein abundance or the phosphorylation level of the target protein was calculated.

### 2.2. IPEC-J2 Cell Experiment

#### 2.2.1. Cell Culture and Treatments

The porcine small intestine epithelial cells (IPEC-J2) were generously provided by Prof. Daiwen Chen from Sichuan Agricultural University in Ya’an, China. Culture the IPEC-J2 cells using Dulbecco’s Modified Eagle Medium (DMEM) medium supplemented with 10% fetal bovine serum (FBS). The cells were seeded at a density of 1 × 10^6^ in a 6-well plate and cultured in a humidified cell incubator maintained at 37 °C with 5% CO_2_. When the cells in the plate reached 60% to 70% confluence, the growth medium was replaced with the treatment medium. In order to investigate the optimal concentration of GLU for IPEC-J2 treatment, culture media containing different concentrations (0, 10, 50, 100, 500, 1000, 5000, 10,000 μg/mL) of GLU were used to culture the cells. In addition, 40 μg/mL lipopolysaccharide (LPS) was added to the culture medium to investigate the effect of GLU in alleviating oxidative stress [30]. To determine if the FOXO1 pathway is involved in the regulation of the intestinal barrier by GLU in piglets, the FOXO1 pathway was blocked using the inhibitor AS1842856 (MCE, Monmouth Junction, NJ, USA).

#### 2.2.2. Cell Viability

IPEC-J2 cells were cultured in 96-well plates at a density of 3 × 10^5^ cells/well. After treatment, the cells were incubated with the Cell Counting Kit-8 (CCK8) (Beyotime, Shanghai, China) for 3 h. Cell viability was then determined by measuring the optical density (OD) at 450 nm using a microplate reader (Multiskan FC, Thermo-Fisher, Rockford, IL, USA).

#### 2.2.3. Measurement of Intracellular ROS Levels

Intracellular ROS levels in IPEC-J2 cells were quantified using the Reactive Oxygen Species Assay Kit (Beyotime, Shanghai, China). After the experimental treatments, the cells were incubated with 10 μM 2′,7′-dichlorodihydrofluorescein diacetate (DCFH-DA), a fluorescent probe for ROS detection, in the dark for 30 min. Subsequently, cells were harvested from each group and subjected to flow cytometric analysis, with data statistically analyzed using FlowJo_V10.9.0 software (Stanford, CA, USA). For further validation, parallel samples were prepared and incubated with an Intracellular ROS Detection Kit (Sigma-Aldrich, WI, USA) for 1 h. ROS (red) fluorescence intensity was observed under a Nikon Eclipse Ti-E upright fluorescence microscope (Nikon, Tokyo, Japan), and the intracellular ROS fluorescence intensity was statistically analyzed using Image J Pro Plus 6.0 software.

#### 2.2.4. Immunofluorometric Assay

After the experimental treatments, the IPEC-J2 cells were first fixed using 4% paraformaldehyde for 15 min. Subsequently, they were permeabilized with 0.1% Triton X-100 and then blocked with 5% BSA for a duration of 30 min. Cells were then incubated overnight at 4 °C with the primary antibody against FOXO1 diluted to 1:200 (Cell Signaling Technology, Danvers, MA, USA). After PBST washes, the cells were incubated with a secondary antibody conjugated to Alexa Fluor 488 (Abcam, Cambridge, UK) for 2 h at room temperature. Then, DAPI dyes (Beyotime, Shanghai, China) were used locate the nucleus. The cells were visualized using a Nikon Eclipse Ti-E inverted fluorescence microscope (Nikon, Tokyo, Japan), and finally, the fluorescence intensity was analyzed with Image J Pro Plus 6.0 software.

#### 2.2.5. Western Blot Analysis of IPEC-J2 Cells

The total protein of IPEC-J2 cells (adding 0.5 mL lysis buffer per well) was extracted with a mixture of RIPA lysis buffer, and the specific experimental procedures for Western blot analysis were the same as described in Section 2.1.12.

### 2.3. Statistical Analysis

Data from each experimental group were analyzed on a per-pen basis. Statistical evaluations were carried out using GraphPad Prism version 8.0 (GraphPad Software, San Diego, CA, USA), using one-way analysis of variance (ANOVA), and post hoc analysis was performed based on the least significant difference. Findings are reported as the mean ± standard error of the mean (SEM); 0.01 < *p* ≤ 0.05 indicates statistically significant differences (*), and *p* ≤ 0.01 indicates a statistically very significant difference (**).

## 3. Results

### 3.1. The Effects of GLU on the Growth Performance of Piglets

Growth performance and diarrhea rates of piglets are shown in Table 3. The ADG, ADFI, and final body weights of piglets in the GLU group were significantly higher than those in the CON group (*p* < 0.05), whereas the diarrhea rate of piglets in the GLU group was significantly lower than that in the CON group (*p* < 0.05). There was no significant difference in the feed conversion rate (G/F) between the CON and GLU groups of piglets (*p* > 0.05).

### 3.2. The Effects of GLU on the Serum Biochemical Parameters of Piglets

**Figure 1:**
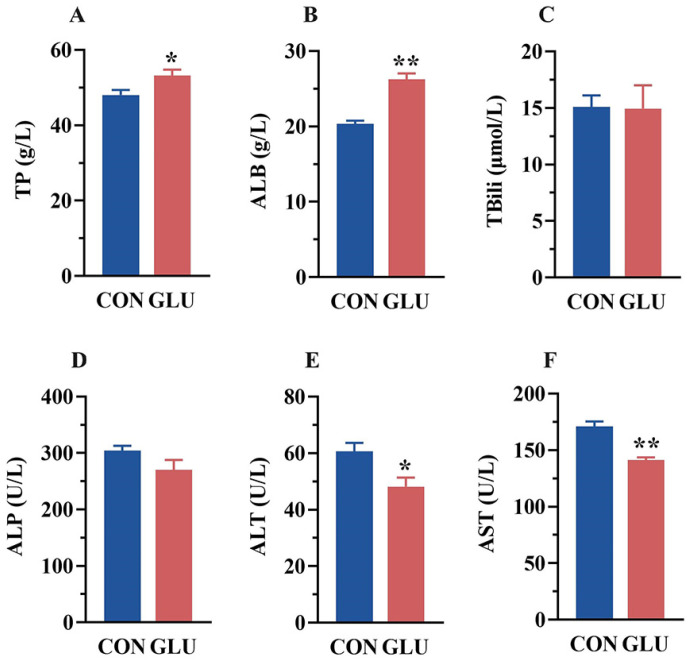
Concentrations of biochemical indicators in the serum of piglets. (**A**) The concentrations of total protein (TP); (**B**) albumin (ALB); (**C**) total bilirubin (TBili); (**D**) alkaline phosphatase (ALP); (**E**) alanine aminotransferase (ALT); (**F**) aspartate aminotransferase (AST). Values are expressed as the mean ± SEM (*n* = 6). *, 0.01 < *p* ≤ 0.05, **, *p* ≤ 0.01.

The serum biochemical parameters of piglets are shown in Figure 1. Compared with the CON group, the serum levels of TP and ALB were increased, whereas the serum levels of ALT and AST were decreased in the GLU group (*p* < 0.05). There was no significant difference in TBili and ALP between the CON and GLU groups of piglets (*p* > 0.05).

**Figure 2:**
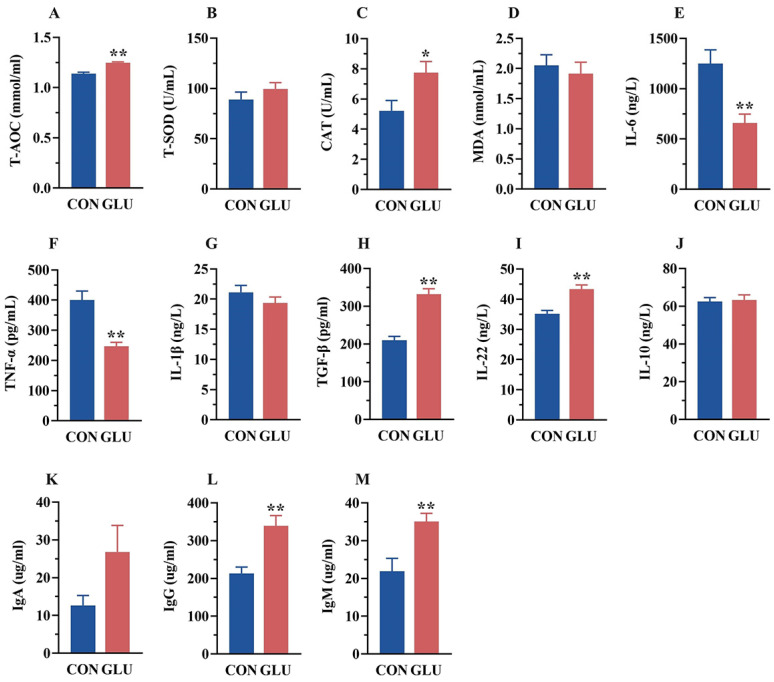
Effects of dietary GLU supplementation on antioxidant status and immune-inflammatory level in the serum of piglets. (**A**–**D**) Antioxidative and oxidative indicators in the serum; (**E**–**J**) concentration of inflammatory factors in the serum; (**K**–**M**) immunoglobulin concentration in the serum. Values are presented as the mean ± SEM (*n* = 6). *, 0.01 < *p* ≤ 0.05, **, *p* ≤ 0.01.

As shown in Figure 2, the serum T-AOC and CAT activities in the GLU group were significantly higher than those in the CON group (*p* < 0.05). Meanwhile, the serum concentrations of IL-6 and TNF-α in the GLU group (*p* < 0.05), whereas the concentrations of TGF-β and IL-22, were significantly higher than those in the CON group (*p* < 0.05). Compared with the CON group, the GLU group showed a significant increase in IgG and IgM concentrations (*p* < 0.05).

### 3.3. The Effects of GLU on the Intestinal Permeability of Piglets

**Figure 3:**
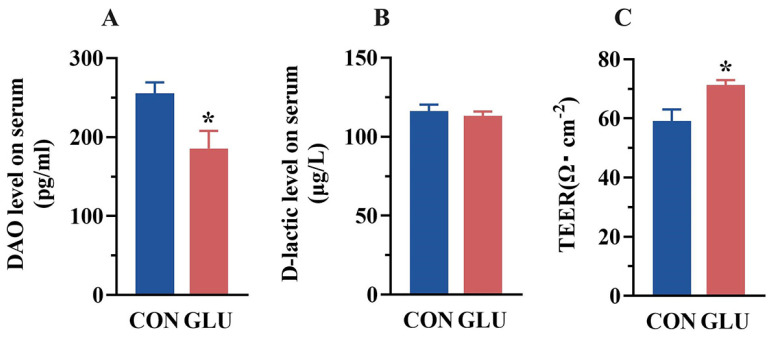
Effect of dietary GLU supplementation on intestinal permeability of piglets. (**A**) DAO, diamine oxidase; (**B**) D-lactic, D-lactic acid; (**C**) transmembrane resistance values. Values are presented as the mean ± SEM (*n* = 6). *, 0.01 < *p* ≤ 0.05.

The concentrations of DAO and D-lactic acid in piglet serum are shown in Figure 3A,B. The DAO concentration in the serum of piglets in the GLU group was significantly lower than that in the CON group (*p* < 0.05). There was no significant difference in the concentration of D-lactic acid in the serum of piglets between the CON group and the GLU group (*p* > 0.05). Compared with the CON group (Figure 3C), the TEER value of piglets in the GLU group was significantly increased (*p* < 0.05).

### 3.4. The Effects of GLU on the Intestinal Morphology in Piglets

**Figure 4:**
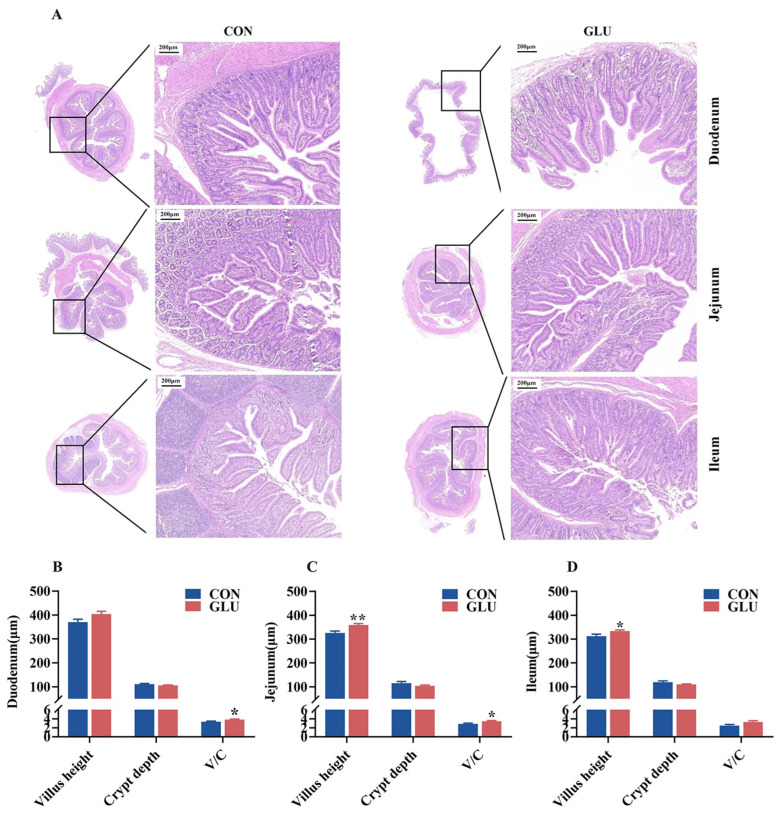
Effect of dietary GLU supplementation on intestinal morphology in piglets. (**A**) H&E staining of piglet intestine; (**B**–**D**) analysis of villus height and crypt depth. CON, control group; GLU, glucuronolactone group. Values are presented as the mean ± SEM (*n* = 6). *, 0.01 < *p* ≤ 0.05, **, *p* ≤ 0.01.

The morphology of the piglet small intestine, encompassing the villus height and crypt depth, is shown in Figure 4. The results of H&E staining revealed a condition of shortened and occasionally fractured villi in the CON group, whereas the GLU group exhibited a more intact and tightly packed villus structure. The villus heights in both the jejunum and ileum of piglets in the GLU group were significantly higher than those in the CON group, and the V/C ratios in both the duodenum and jejunum of piglets in the GLU group were significantly higher than those in the CON group (*p* < 0.05).

### 3.5. The Effects of GLU on the Expression of Intestinal Tight Junction Proteins in Piglets

**Figure 5:**
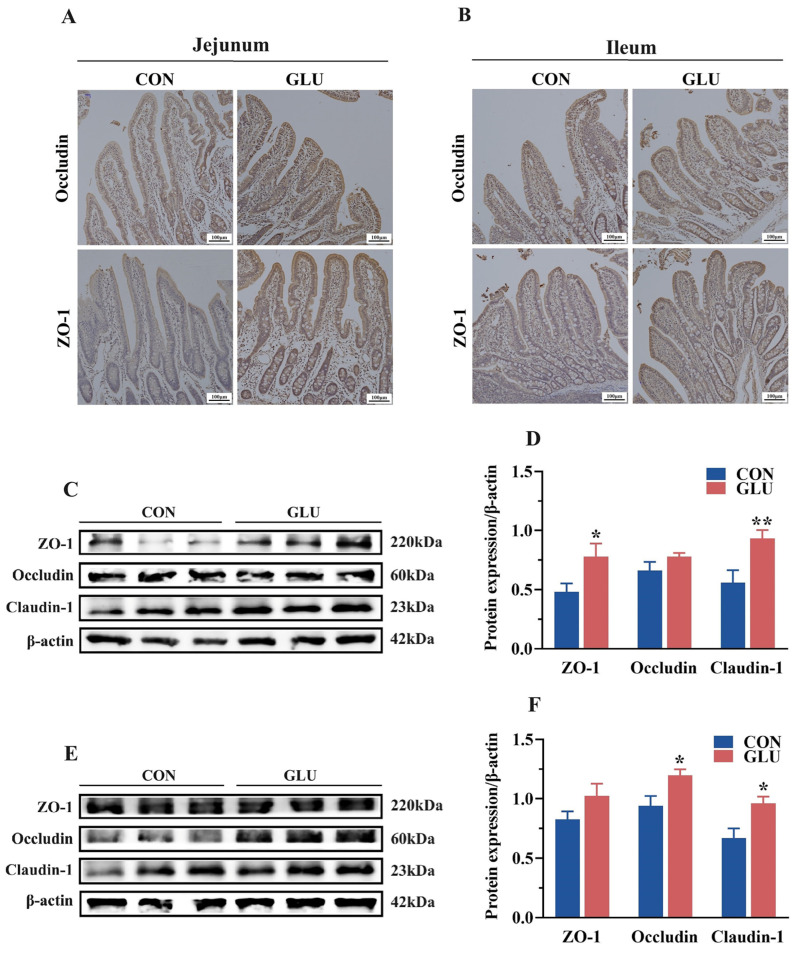
Effects of dietary GLU supplementation on the expression of tight junctions in the intestine. (**A**,**B**) Immunoreactivity of tight junctions in the jejunum (**A**) and ileum (**B**); (**C**–**F**) Western blot analysis of tight junctions in the jejunum (**C**,**D**) and ileum (**E**,**F**). Values are presented as the mean ± SEM (*n* = 6). *, 0.01 < *p* ≤ 0.05, **, *p* ≤ 0.01.

The expression levels of tight junctions in the jejunum and ileum are shown in Figure 5. An immunohistochemical analysis revealed the significantly heightened positive expression of ZO-1 and Occludin in the GLU group compared to that in the CON group (Figure 5A,B). Western blot analyses showed that the relative protein abundance of ZO-1 and Claudin-1 in the jejunum and Claudin-1 and Occludin in the ileum in the GLU group were significantly higher than those in the CON group (Figure 5C–F) (*p* < 0.05).

### 3.6. The Effects of GLU on the Relative Expression of Intestinal Mucin and Porcine β-Defensin in Piglets

**Figure 6:**
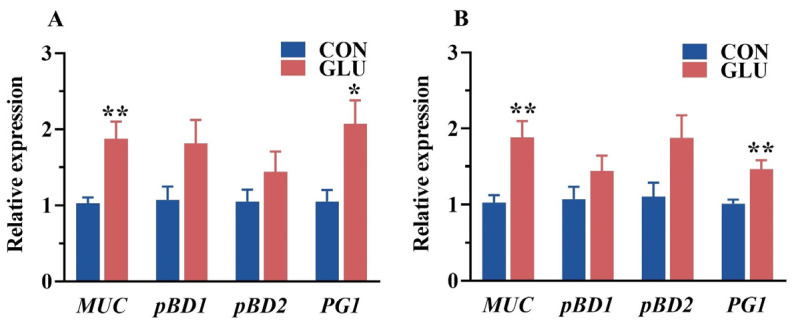
Effects of dietary GLU supplementation on the relative expression of mucins and porcine beta defensins in the intestines of piglets. (**A**) Jejunum; (**B**) ileum. *MUC*, mucin; *pBD*, porcine beta defensins; *PG1*, Protegrin-1. Values are presented as the mean ± SEM (*n* = 6). *, 0.01 < *p* ≤ 0.05, **, *p* ≤ 0.01.

The relative expression of porcine beta defensins and mucins in the jejunum and ileum of piglets is shown in Figure 6. The relative expression of *MUC* and *PG1* in the jejunum and ileum in the GLU group was significantly higher than those in the CON group (*p* < 0.05).

### 3.7. The Effects of GLU on the Antioxidant Capacity, Immunoglobulins, and Inflammatory Factors in the Intestine of Piglets

**Figure 7:**
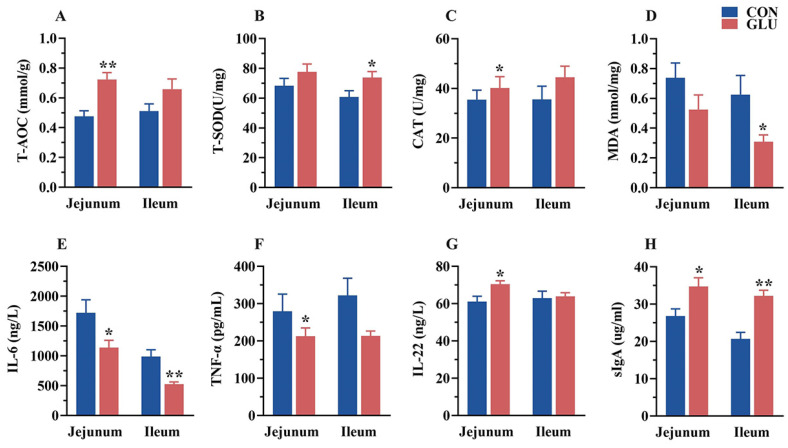
Effects of dietary GLU supplementation on antioxidant status and immune-inflammatory level in the intestines of piglets. (**A**–**D**) Antioxidative and oxidative indicators concentration; (**E**–**G**) inflammatory factor concentration; (**H**) secretory Immunoglobulin A. Values are presented as the mean ± SEM (*n* = 6). *, 0.01 < *p* ≤ 0.05, **, *p* ≤ 0.01.

The concentrations of antioxidant capacity, immunoglobulins, and inflammatory factors in the jejunum and ileum of piglets are shown in Figure 7. In the jejunum, the concentrations of T-AOC, CAT, IL-22, and sIgA in the GLU group were significantly higher than those in the CON group, whereas the concentrations of IL-6 and TNF-α were significantly lower than those in the CON group (*p* < 0.05). In the ileum, the concentrations of T-SOD and sIgA in the GLU group were significantly higher than those in the CON group, whereas the concentrations of MDA and IL-6 were significantly lower than those in the CON group (*p* < 0.05).

### 3.8. The Effects of GLU on the Microorganisms in the Contents of the Ileum and Colon in Piglets

A total of 1,555,530 effective sequences were generated from 24 content samples (two groups, *n* = 6), with an average of 64,814 sequences per sample, and 14,336 OTUs were obtained for further analysis. The sequencing data are available online at the National Genomics Data Centre (Beijing, China), under accession number CRA017814.

**Figure 8:**
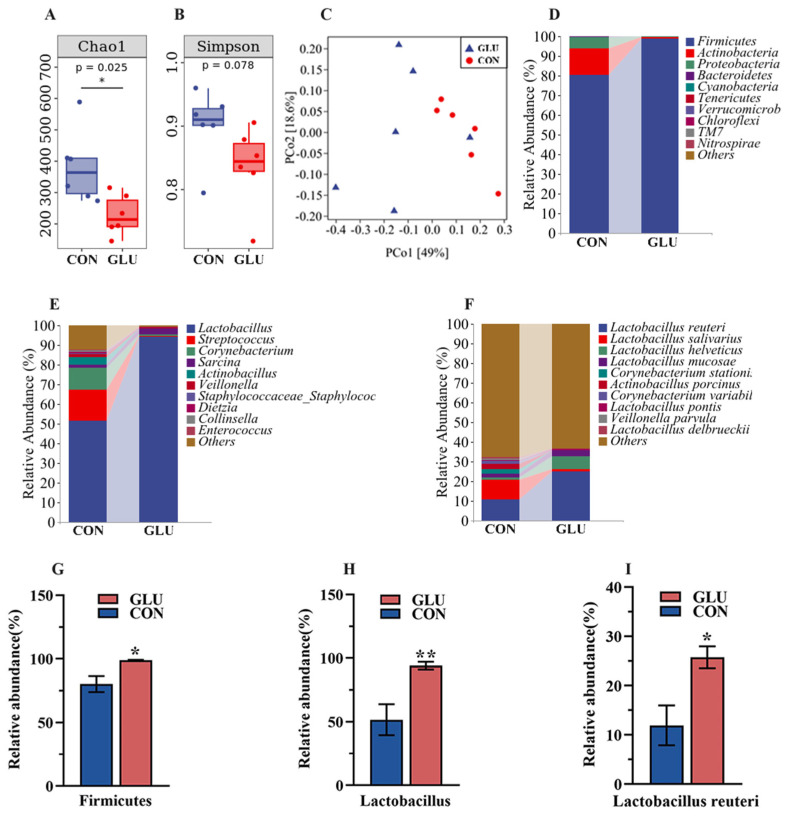
Effects of dietary GLU supplementation on the ileal microbiota of piglets. (**A**,**B**) Alpha diversity; (**C**) beta diversity; (**D**–**I**) relative abundance of microbiota at the phylum, genus, and species levels. Values are presented as the mean ± SEM (*n* = 6). *, 0.01 < *p* ≤ 0.05, **, *p* ≤ 0.01.

The analysis results of microbiota in the ileum of piglets are shown in Figure 8. The three major bacterial phyla in the ileal contents of piglets were Firmicutes (80.26% or 98.79%), Actinobacteria (13.49% or 1.01%), and Proteobacteria (5.95% or 0.07%), accounting for over 99% of the total bacterial community. Alpha diversity results showed a significantly lower Chao 1 index in the GLU group than in the CON group (*p* < 0.05), whereas the Simpson index did not show a statistically significant difference (Figure 8A,B). For the beta diversity, a Principal Coordinates Analysis (PCoA) revealed a distinct separation trend between GLU group and CON group (Figure 8C). At the phylum level, the relative abundance of Firmicutes in the GLU group was significantly higher than that in the CON group (*p* < 0.05). At the genus level, the relative abundance of Lactobacillus in the GLU group was significantly higher than that in the CON group (*p* < 0.05). At the species level, *Lactobacillus reuteri* was identified as the primary driver of change, with its abundance surging from 11.93% to 25.74% (Figure 8D–I) (*p* < 0.05).

**Figure 9:**
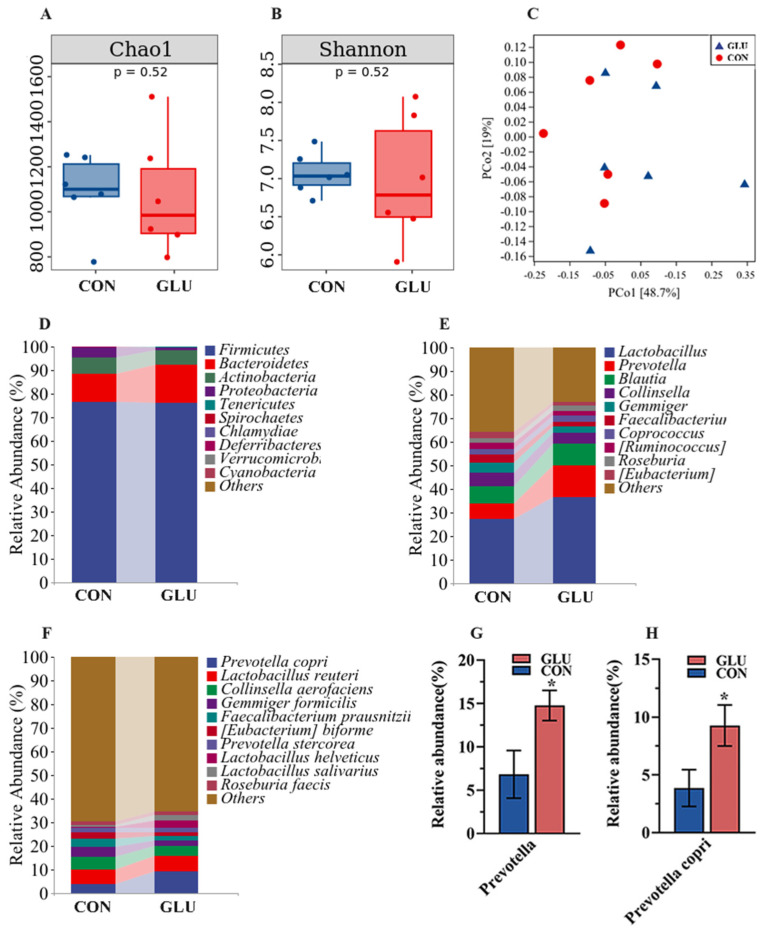
Effects of dietary GLU supplementation on the colonic microbiota of piglets. (**A**,**B**) Alpha diversity; (**C**) beta diversity; (**D**–**H**) relative abundance of microbiota at the phylum, genus, and species levels. Values are presented as the mean ± SEM (*n* = 6). *, 0.01 < *p* ≤ 0.05.

The analysis results of microbiota in the colons of piglets are shown in Figure 9. Three major bacterial phyla in the colonic contents of piglets were Firmicutes (76.59% or 76.00%), Bacteroidetes (11.83% or 16.46%), and Actinobacteria (6.98% or 5.81%), accounting for over 95% of the total bacterial community. Alpha diversity within the colon microbiome disclosed no statistically significant disparities in either the Chao 1 or Shannon indices between the GLU and CON group (Figure 9A,B). For the beta diversity, the PCoA analysis suggested that there was no marked differentiation between the GLU group and CON group (Figure 9C). At the genus level, the Prevotella abundance in GLU group was significantly higher than in the CON group. At the species level, *Prevotella copri* emerged as the predominant species driving this shift, with its prevalence escalating from 3.87% to 9.28% (Figure 9D–H) (*p* < 0.05).

### 3.9. Network Pharmacological Analysis Among GLU, Oxidative Stress, and Inflammatory

**Figure 10:**
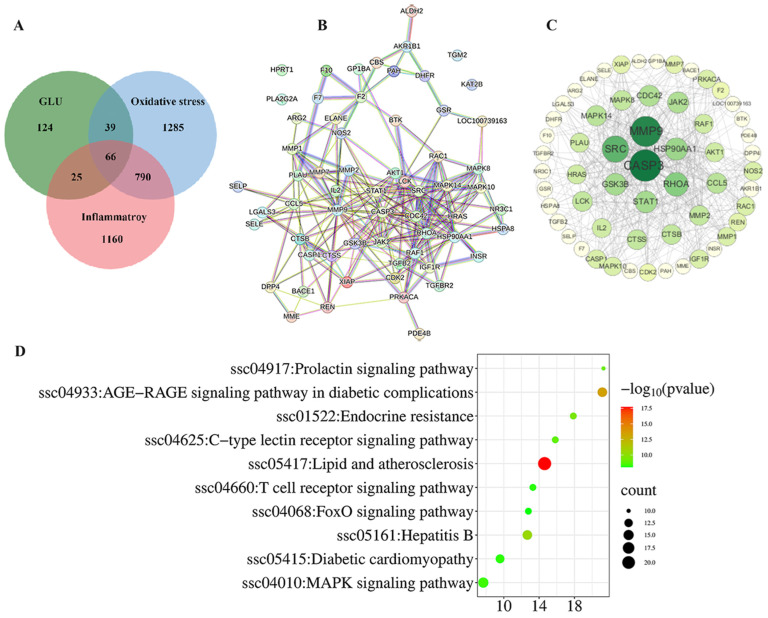
Network pharmacological analysis among GLU, oxidative stress, and inflammation. (**A**–**C**) Intersection analysis between GLU targets and the disease targets of oxidative stress and inflammatory, as well as screening of core targets; (**D**) the top 10 KEGG pathways.

The network pharmacology analysis among GLU, oxidative stress, and inflammation is illustrated in Figure 10. A total of 66 potential targets for GLU to alleviate oxidative stress and inflammation were identified. These targets were inputted into the STRING database with the Organisms parameter set to Sus scrofa to construct the protein–protein interaction (PPI) network (Figure 10A). Additionally, Cytoscape 3.10 software was employed to visualize the PPI network based on this information. Following the topological analysis of the network, the core target screening criterion was set to a degree threshold of >26. Seven core targets were identified, including CASP3, MMP9, SRC, RHOA, HSP90AA1, GSK3B, and STAT1(Figure 10B,C). Furthermore, the functional roles of the 66 potential targets were predicted through GO and KEGG enrichment analysis (Figure 10D). The top 10 signaling pathways were identified, with the “MAPK signaling pathway” and the “FOXO signaling pathway” highlighted as the key pathways through which GLU regulates oxidative stress and inflammation in piglets.

**Figure 11:**
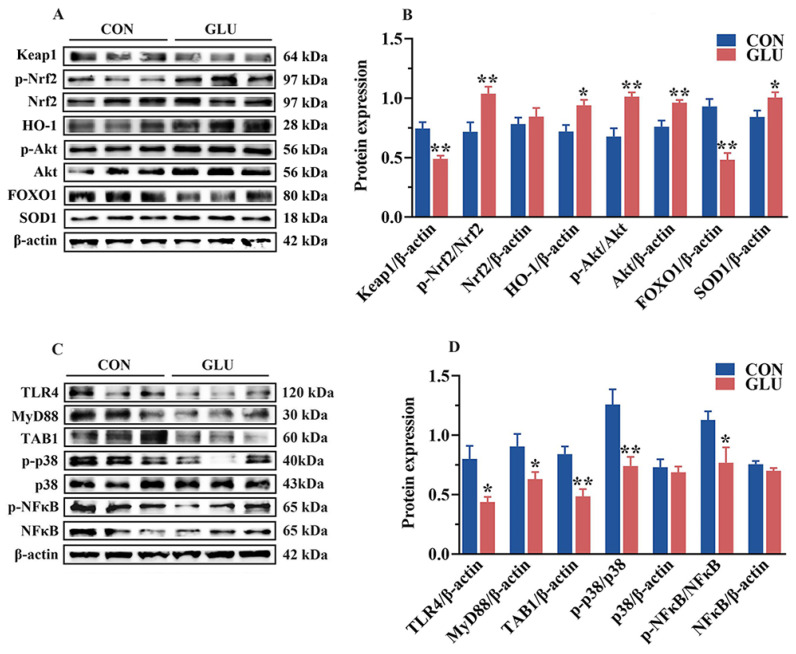
Effects of dietary GLU supplementation on activation of the TLR4/MAPK and Nrf2/FOXO1 signaling pathways in the jejunum of piglets. (**A**,**B**) Western blot analysis of the Nrf2/Akt/FOXO1 signaling pathways; (**C**,**D**) Western blot analysis of the TLR4/MAPK signaling pathways. *, 0.01 < *p* ≤ 0.05, **, *p* ≤ 0.01.

Next, Western blot analysis was performed to validate the functions of these pathways (Figure 11). In the jejunum of piglets, the phosphorylation of Akt and Nrf2 and the relative abundance of HO-1, Akt, and SOD1 proteins in the GLU group were significantly higher than those in the CON group (*p* < 0.05). However, the relative abundance of Keap1 and FOXO1 in the GLU group was significantly lower than that in the CON group (*p* < 0.05) (Figure 11A,B). In addition, compared to the CON group, the relative abundances of TLR4, MyD88, and TAB1 proteins, as well as the phosphorylation of p38 and NF-κB, were significantly decreased in the GLU group (Figure 11C,D), while there was no significant difference in the relative abundance of Nrf2, p38, and NF-κB (*p* > 0.05).

### 3.10. Effect of GLU Treatment on LPS-Challenged IPEC-J2 Cells

**Figure 12:**
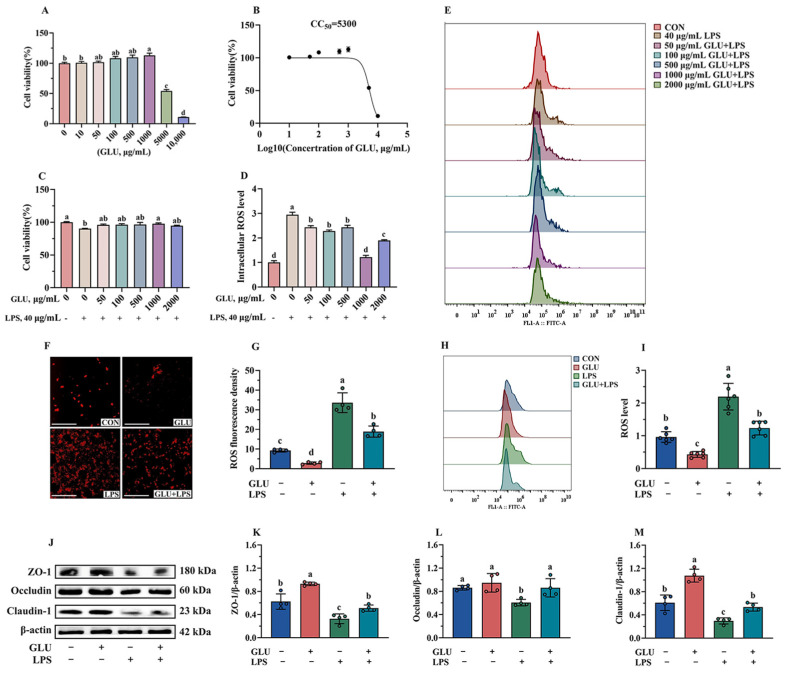
Effect of GLU treatment on LPS-challenged IPEC-J2 cells. (**A**) IPEC-J2 cell viability; (**B**) CC50 value of GLU; (**C**) effect of GLU treatment on cell viability in LPS-challenged IPEC-J2 cells; (**D**,**E**) effect of GLU treatment on the ROS level in LPS-challenged IPEC-J2 cell; (**F**–**I**) the ROS level in IPEC-J2 cells, scale bar 500 μm; (**J**–**M**) Western blot analysis of the tight junctions. Values are expressed as the mean ± SEM (*n* = 4). Different letters (abcd) indicate significant differences.

As shown in Figure 12A, GLU treatment showed no negative effect on cell viability when its treatment concentration did not exceed 1000 μg/mL. However, GLU treatment at concentrations of 5000 μg/mL and 10,000 μg/mL significantly reduced the cell viability (*p* < 0.05). In addition, the CC_50_ of GLU was estimated to be 5300 μg/mL using GraphPad analysis (Figure 12B). Notably, compared with the control group, treatment with 1000 μg/mL GLU significantly increased the cell viability of LPS-challenged IPEC-J2 cells (*p* < 0.05) (Figure 12C). Meanwhile, treatment with 1000 μg/mL GLU significantly reduced the accumulation of ROS in LPS-challenged IPEC-J2 cells (*p* < 0.05), and there was no significant difference compared to the blank control group (Figure 12D,E). Based on these results, GLU at a concentration of 1000 μg/mL was selected for subsequent experiments.

As shown in Figure 12F,I, the results of immunofluorescence and flow cytometry analysis indicated that GLU treatment significantly reduced ROS accumulation, while LPS exposure significantly increased ROS accumulation in IPEC-J2 cells (*p* < 0.05). Notably, GLU treatment significantly reduced ROS accumulation in LPS-challenged IPEC-J2 cells (*p* < 0.05). Consistently, GLU treatment significantly increased the relative protein abundance of Claudin-1, Occludin, and ZO-1 compared to the blank control group (*p* < 0.05), while LPS exposure significantly decreased Claudin-1, Occludin, and ZO-1 (*p* < 0.05). The co-administration of GLU and LPS significantly increased the relative protein abundance of Claudin-1, Occludin, and ZO-1 in IPEC-J2 cells compared to LPS exposure (*p* < 0.05).

**Figure 13:**
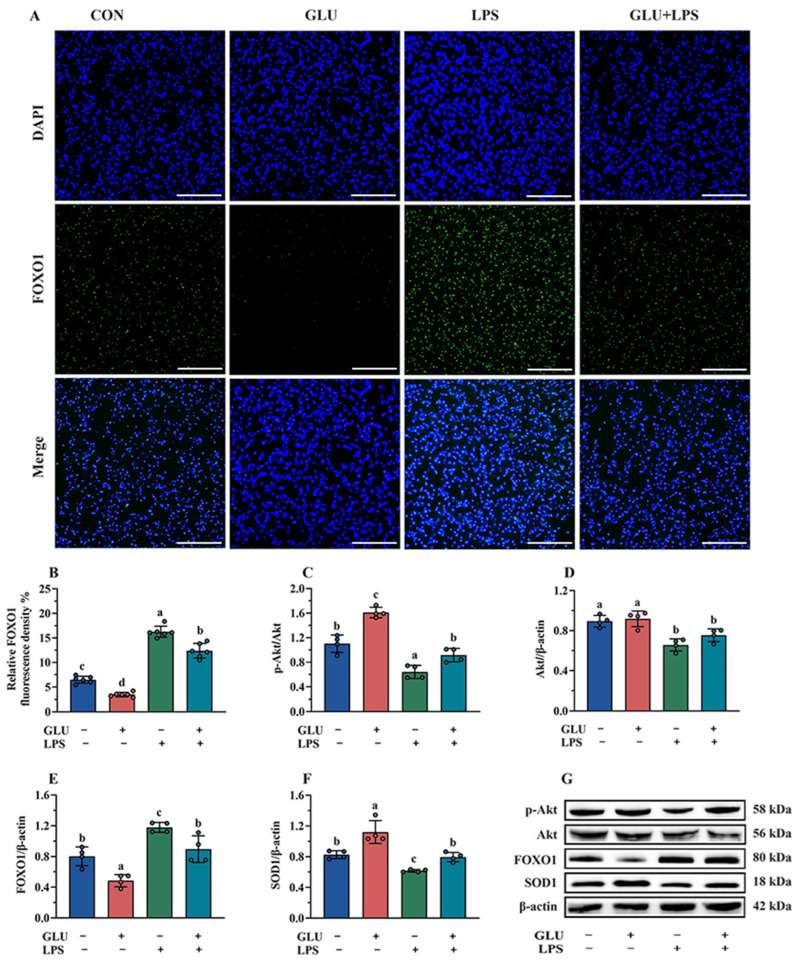
Effect of GLU treatment on the Akt/FOXO1/SOD1 pathway in LPS-challenged IPEC-J2 cells. (**A**,**B**) The immunofluorescence microscopy of FOXO1, scale bar 500 μm; (**C**–**G**) Western blot analysis of the Akt/FOXO1/SOD1 pathway. Values are expressed as the mean ± SEM (*n* = 4). Different letters (abcd) indicate significant differences.

As shown in Figure 13, the results of immunofluorescence and Western blotting indicated that GLU treatment significantly decreased FOXO1 expression in IPEC-J2 cells compared to the blank control group (*p* < 0.05), while LPS exposure significantly increased FOXO1 expression (*p* < 0.05). Importantly, GLU treatment significantly reduced the effect of LPS exposure on FOXO1 expression in IPEC-J2 cells (*p* < 0.05). However, GLU treatment significantly increased Akt phosphorylation and Akt relative protein abundance in IPEC-J2 cells compared to the blank control group (*p* < 0.05), while LPS exposure significantly decreased Akt phosphorylation and the Akt relative protein abundance (*p* < 0.05). Moreover, GLU treatment inhibited the decrease in Akt phosphorylation caused by LPS exposure in IPEC-J2 cells (*p* < 0.05).

### 3.11. Effect of GLU Treatment and FOXO1 Pathway Inhibition on ROS Accumulation in IPEC-J2 Cells

**Figure 14:**
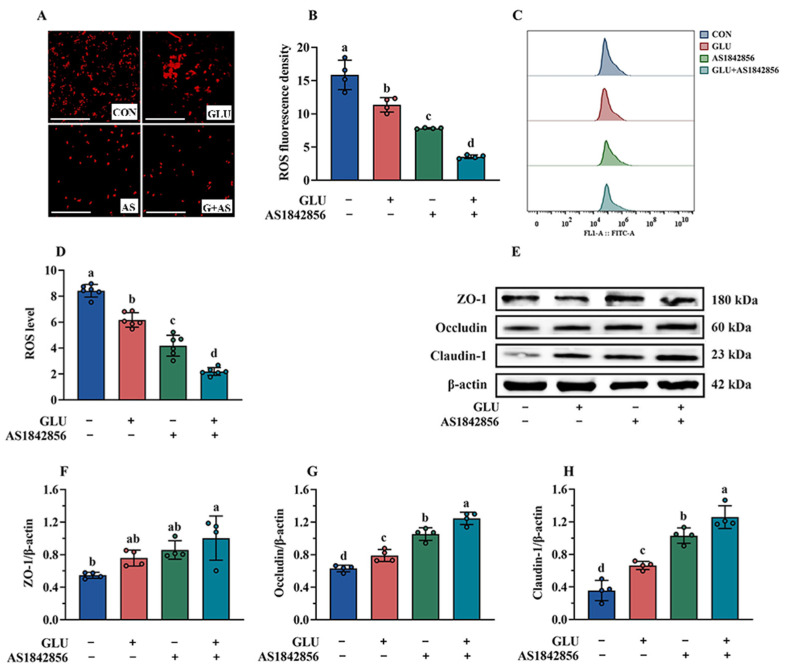
Effect of GLU treatment on the ROS levels and tight junctions in AS1842856-challenged IPEC-J2 cells. (**A**–**D**) The ROS levels in IPEC-J2 cells, scale bar 500 μm; (**E**–**H**) Western blot analysis of the tight junctions. Values are expressed as the mean ± SEM (*n* = 4). Different letters (abcd) indicate significant differences.

As shown in Figure 14A–D, the results of immunofluorescence and flow cytometry analysis indicated that AS1842856 treatment significantly decreased ROS accumulation in IPEC-J2 cells compared to the blank control group (*p* < 0.05). Compared with the AS1842856 treatment group, the co-administration of GLU and AS1842856 significantly reduced ROS accumulation in IPEC-J2 cells (*p* < 0.05). Consistently, AS1842856 treatment significantly increased the relative protein abundance of Claudin-1, Occludin, and ZO-1 compared to the blank control group (*p* < 0.05) (Figure 14E–H), and also, the co-administration of GLU and AS1842856 significantly increased the relative protein abundance of Claudin-1, Occludin, and ZO-1 in IPEC-J2 cells compared to the AS1842856 treatment (*p* < 0.05).

**Figure 15:**
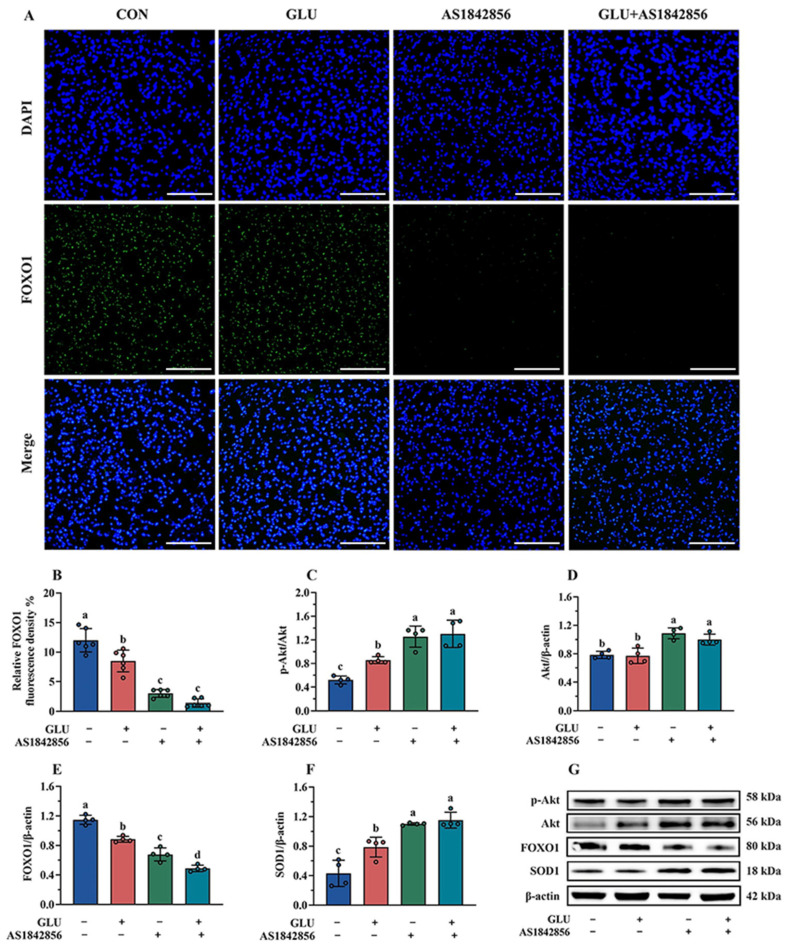
Effect of GLU treatment on the Akt/FOXO1/SOD1 pathway in AS1842856-challenged IPEC-J2 cells. (**A**,**B**) The immunofluorescence microscopy of FOXO1 in IPEC-J2 cells, scale bar 500 μm; (**C**–**G**) Western blot analysis of the Akt/FOXO1/SOD1 pathway. Values are expressed as the mean ± SEM (*n* = 4). Different letters (abcd) indicate significant differences.

The immunofluorescence and Western blot results of the FOXO1 pathway in IPEC-J2 cells are shown in Figure 15. As shown in Figure 15A,B, GLU treatment significantly decreased the expression of FOXO1 in IPEC-J2 cells compared to the blank control group, and also, AS1842856 treatment significantly reduced the expression of FOXO1 (*p* < 0.05). Compared with the AS1842856 treatment group, the co-administration of GLU and AS1842856 significantly decreased the expression of FOXO1 in IPEC-J2 cells (*p* < 0.05). Furthermore, GLU treatment significantly increased SOD1 and reduced FOXO1 protein abundance in IPEC-J2 cells compared to the blank control group, and also, AS1842856 treatment significantly increased SOD1 and reduced FOXO1 protein abundance (*p* < 0.05) (Figure 15C–G). Compared with the AS1842856 treatment group, the co-administration of GLU and AS1842856 significantly the reduced FOXO1 and increased SOD1 abundance in IPEC-J2 cells (*p* < 0.05). However, GLU treatment significantly increased the Akt phosphorylation in IPEC-J2 cells compared to the blank control group, also AS1842856 treatment significantly increased the Akt phosphorylation and Akt relative protein abundance (*p* < 0.05). Compared with the AS1842856 treatment group, the co-administration of GLU and AS1842856 significantly increased the Akt phosphorylation in IPEC-J2 cells (*p* < 0.05).

## 4. Discussion

The intestine is a critical site for nutrient digestion and absorption in piglets. Maintaining the integrity of intestinal barrier is essential for unlocking the growth potential of piglets [31]. Numerous studies have shown that oxidative stress induced by weaning stress impairs the integrity of the intestinal barrier in piglets, which is considered to be a key factor contributing to post-weaning growth retardation in piglets [32,33]. Therefore, employing nutritional strategies to alleviate intestinal oxidative stress in post-weaning piglets is crucial for improving their growth performance. Previous research has indicated that certain antioxidants play a positive role in maintaining the intestinal function and improving growth performance in weaned piglets [13,34,35]. GLU is an intermediate metabolite of glucose metabolism that is rapidly converted into glucuronic acid upon entering the body [16,17]. Glucuronic acid is a potent antioxidant known for its ability to scavenge free radicals in cells, thereby mitigating oxidative damage [16,18,19]. In the present study, we found that dietary supplementation with 200 mg/kg GLU significantly increased the ADG, ADFI, and final body weights of piglets, indicating that GLU has a positive impact on improving the growth performance of piglets. Additionally, dietary GLU supplementation markedly reduced the diarrhea rate in piglets, underscoring its importance in improving intestinal health. At present, there are no studies available on the application of GLU in livestock production, highlighting the novelty and potential significance of this research.

In order to evaluate the beneficial effects of GLU on the intestinal health in piglets, this study firstly measured the concentrations of D-lactate and diamine oxidase (DAO) in piglet serum. Serum D-lactate levels and DAO activity are commonly used as biomarkers to monitor the extent of intestinal barrier injury [36,37,38]. D-lactic acid is a specific end-product of bacterial metabolism in the intestine [39], while DAO is an intracellular enzyme primarily synthesized primarily by mammalian intestinal epithelial cells [40]. When the intestinal epithelial barrier is destroyed, D-lactic acid and DAO leak or are released into the circulation, resulting in abnormally elevated serum concentrations [36,37,38]. In this study, GLU supplementation significantly reduced serum DAO activity in piglets, indicating that GLU alleviates the damage to intestinal epithelial cells caused by weaning stress. However, no significant difference was observed in serum D-lactate levels between the two groups, which may reflect variations in the extent of intestinal epithelial damage. Moreover, transepithelial electrical resistance (TEER) is a commonly used indicator for assessing intestinal permeability in piglets [41]. Greater intestinal damage leads to increased permeability and lower TEER values [42]. This study found that GLU supplementation significantly increased the TEER value, suggesting that GLU reduced intestinal permeability in piglets. Therefore, dietary supplementation with GLU exerts beneficial effects in mitigating the intestinal damage induced by weaning stress in piglets.

The intestinal epithelial barrier is a crucial component of the intestinal barrier in piglets [43,44]. The intestinal epithelial barrier is composed of tight junctions between intestinal epithelial cells, for which the function is largely maintained and regulated by these tight junctions [45]. Tight junctions are directly or indirectly anchored to the actin-based cytoskeleton, thereby forming a selective permeable barrier [46]. Previous studies have demonstrated that tight junctions consist of several unique proteins [47,48], including the Claudin families, Occludin, and intracellular linker proteins, such as ZOs. The functionality of the intestinal epithelial barrier is closely linked to the distribution and expression levels of tight junction proteins. However, weaning stress can disrupt the intestinal epithelial barrier in piglets by inhibiting the expression of tight junction proteins [23]. In this study, an immunohistochemical analysis revealed that dietary supplementation with GLU significantly increased the protein abundance of Occludin and ZO-1 at the apical regions of villi in the jejunum and ileum of weaned piglets. Consistently, piglets fed a diet supplemented with GLU exhibited higher relative protein abundances of Claudin-1, Occludin, and ZO-1 in the intestine. Similarly, this study found that GLU significantly improved the relative protein abundances of Claudin-1, Occludin, and ZO-1 in IPEC-J2 cells. Furthermore, the present study showed that GLU had a beneficial effect on improving the villus morphology in the small intestine in piglets. These findings suggest that GLU may maintain the function of the intestinal epithelial barrier by modulating the expression of tight junctions, which may partly partially explain its role in improving gut health in weaned piglets under stress.

The accumulation of reactive oxygen species (ROS) in intestinal epithelial cells due to a redox imbalance is a key intrinsic factor contributing to intestinal barrier dysfunction in piglets [24,35]. To elucidate the underlying mechanism of intestinal barrier dysfunction in weaned piglets, this study analyzed the activities of antioxidant enzymes in the intestinal tissue and serum of piglets. The findings revealed that dietary supplementation with GLU significantly increased the concentrations of T-AOC, T-SOD, and CAT in the intestine, while also enhancing the levels of T-AOC and CAT in the serum of piglets. In IPEC-J2 cells, this study also found that GLU increased the relative protein abundance of SOD1 and reduced ROS accumulation. These results demonstrate the positive effect of GLU in alleviating oxidative stress in piglets. Similarly, a previous study showed that GLU alleviated ochratoxin A (OTA)-induced liver damage in chickens by increasing the concentrations of SOD and GSH-Px in hepatocytes [14]. Intriguingly, some natural antioxidants have also been shown to improve intestinal oxidative stress and barrier functions by increasing the activities of T-AOC, T-SOD, CAT, and GSH-Px while reducing MDA levels in piglet serum, such as resveratrol and taurine [24,35,49]. Therefore, GLU may improve the intestinal health in piglets by enhancing the activity of antioxidant enzymes in intestinal cells, with its mechanism for mitigating intestinal oxidative stress potentially resembling that of natural antioxidants.

Physiologically, disruption of the redox balance is often accompanied by inflammation. The excessive production of cytokines, particularly pro-inflammatory cytokines, such as TNF-α, IL-6, and IL-1β, can directly impair the intestinal epithelial barrier [23]. Therefore, inhibiting the excessive release of pro-inflammatory cytokines is a highly effective strategy for alleviating the intestinal diseases caused by oxidative stress. This study found that dietary supplementation with GLU significantly reduced the concentrations of IL-6 and TNF-α, while increasing the IL-22 concentration in the intestinal tissue. Additionally, a serum analysis revealed a decrease in TNF-α levels, and the IL-22 concentration was increased in piglets. Cytokines play a crucial role in immune and inflammatory responses [23,50,51]. In this study, we observed that GLU supplementation up-regulated the concentration of secretory immunoglobulin A (sIgA) in the jejunum and ileum and increased the levels of IgG and IgM in the serum of piglets. These findings indicate that GLU alleviates inflammation in the small intestine and even the whole body of piglets, while simultaneously enhancing immune functions.

Currently, molecular dynamic simulations based on bioinformatics have been widely recognized as significant for guiding scientific research. To further explore the molecular mechanisms through which GLU alleviates intestinal oxidative stress and inflammation, this study utilized the Traditional Chinese Medicine Systems Pharmacology Database and Analysis Platform (TCMSP) to predict the antioxidant and anti-inflammatory mechanisms of GLU. The prediction results indicate that the FOXO signaling pathway and the MAPK signaling pathway may be key pathways through which GLU regulates oxidative stress and inflammation. FOXO proteins are regulators and well-documented targets involved in the cell cycle, proliferation, apoptosis, metabolism, and oxidative stress responses [52,53]. As the core member of the FOXO protein family, FOXO1 plays an important role in regulating oxidative stress and inflammation [53]. It is known that the transcriptional activity of FOXO1 is regulated by the PI3K/AKT signaling pathway [54,55]. The activation of PI3K–AKT promotes the phosphorylation of FOXO1, blocking the DNA-binding domain of FOXO, inhibiting FOXO1 transcriptional activity, and leading to cell survival [55,56]. In contrast, the FOXO1 dephosphorylation increases its nuclear accumulation/activity, thereby enhancing cell apoptosis and the expression of target genes, such as pro-inflammatory cytokines [53,57]. Consistently, this study found that GLU improved intestinal oxidative stress and inflammation in piglets, while reducing the protein abundance of FOXO1 in the piglet intestinal tissues. These results suggest that GLU may alleviate intestinal oxidative stress and inflammation in piglets by mediating the PI3K/AKT-FOXO1 signaling pathway.

As a master regulatory factor of the cellular antioxidant defense system, nuclear factor erythroid 2-related factor 2 (Nrf2) plays a crucial role in regulating the inflammatory response associated with oxidative-stress-induced organ damage [58]. The activation of Nrf2 helps mitigate oxidative damage to cells, promoting cell growth and survival, whereas the disruption of Nrf2 enhances oxidative-stress-induced inflammatory responses, thereby exacerbating cell damage [59]. Numerous studies have shown that the Nrf2–Akt–FOXO1 signaling network is a critical pathway for regulating intracellular oxidative stress and inflammation [53]. The activation of Nrf2 promotes PI3K/Akt signaling, thereby inhibiting FOXO1 activity in local inflammatory responses [55]. In contrast, lipopolysaccharide induction or Nrf2 deficiency reduces the expression of PI3K/Akt in cells or tissues, increases the transcription of FOXO1, and leads to the enhanced expression of TLR4 pro-inflammatory mediators [55,60]. The TLR4–MAPK signaling pathway is a classic pathway that regulates cellular inflammation and immune responses. Therefore, this study analyzed the activation of Nrf2–Keap1 signaling pathway and TLR4–MAPK signaling pathway in the intestines of piglets. This study found that GLU promotes the activation of the Nrf2–Keap1 signaling pathway while inhibiting the TLR4–MAPK signaling pathway. Additionally, this study observed that GLU increased the activity of antioxidant enzymes and reduced the levels of pro-inflammatory factors in the intestines of piglets. Importantly, in the in vitro experiments, this study demonstrated that GLU inhibited the activity of the FOXO1 protein by promoting the phosphorylation of Akt, which reduced the accumulation of ROS and increased the expression of SOD1, Claudin-1, Occludin, and ZO-1 in IPEC-J2 cells. These results suggest that GLU alleviates intestinal oxidative stress and inflammation in piglets by activating the Nrf2–AKT signaling pathway to suppress FOXO1 transcriptional activity, while simultaneously reducing the secretion of pro-inflammatory factors by inhibiting the activation of the TLR4–MAPK signaling pathway.

In addition, severe disruption of the gut microbiota can induce intestinal inflammation, disrupt the intestinal barrier, and increase the piglet diarrhea rate [61]. Numerous studies have reported that weaning stress is a key factor leading to disruption of the gut microbiota in piglets during weaning [62]. To assess the effect of GLU on intestinal bacterial colonization in piglets, this study employed high-throughput 16S rDNA sequencing technology to analyze the microbiota in the ileum and colon of piglets. The β-diversity analysis of the ileal microbiota revealed a distinct separation between the GLU group and the CON group, suggesting that GLU significantly altered the microbial composition in the ileum of piglets. At the genus level, the relative abundance of Lactobacillus in the GLU group was significantly higher than that in the CON group, with *Lactobacillus reuteri* identified as the primary driver of these changes. Similarly, previous studies have found that a significant increase in the relative abundance of Lactobacilli, especially *Lactobacillus reuteri*, in the intestines of piglets fed an antioxidant diet [24,35]. *Lactobacillus reuteri* exhibits excellent probiotic properties, including positive regulation of the redox state, inflammation, and immune functions of piglets, as well as the competitive inhibition of pathogens [35,63,64]. Moreover, *Lactobacillus reuteri* has been found to be associated with the secretion of mucin and defense peptides in the intestine [65,66]. Intriguingly, this study found that GLU significantly increased the relative expression of *MUC* and *PG1* in the jejunum and ileum of piglets, which is consistent with previous reports. Meanwhile, this study also found that the effect of GLU on the colonic microbiota was significantly weakened, which may be related to the fact that GLU is mainly absorbed in the small intestine. In summary, this study found that GLU increased the abundance of *Lactobacillus reuteri* in the piglet ileum, which has positive implications for improving the intestinal health of piglets.

## 5. Conclusions

**Figure 16:**
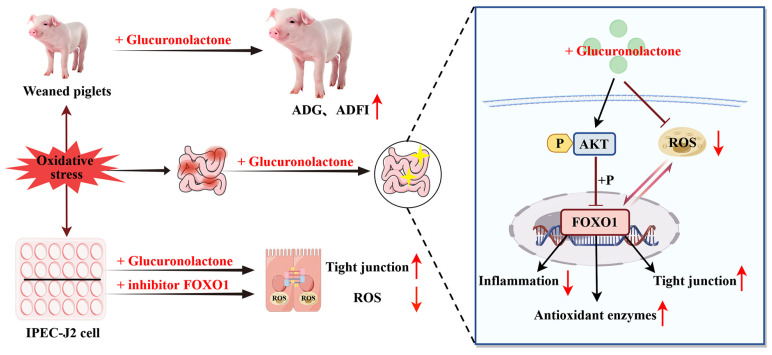
Glucuronolactone restores the intestinal barrier and redox balance partly through the Nrf2/Akt/FOXO1 pathway to alleviate weaning-stress-induced intestinal dysfunction in piglets. The red arrows pointing upward indicates upregulation, while the arrow pointing downward indicates downregulation.

This study found that dietary supplementation with 200 mg/kg GLU increased the ADG, ADFI, and final body weights of piglets, while reducing the diarrhea rate. Mechanistically, GLU restores the intestinal barrier and redox balance partly through the Nrf2/Akt/FOXO1 pathway to alleviate weaning-stress-induced intestinal dysfunction in piglets (Figure 16). Meanwhile, GLU reduces the secretion of pro-inflammatory cytokines by inhibiting the activation of the TLR4/MAPK signaling pathway, thereby reducing intestinal inflammation. Moreover, GLU increases the relative abundance of *Lactobacillus reuteri* in the ileum of piglets and improves the composition of the gut microbiota.

## Figures and Tables

**Table 1:** The composition of ingredients and nutrient levels in the basal diet.

Ingredient	%	Nutrient Levels ^2^	
Corn	41.21	NE, kcal/kg	2600.00
Enzyme-treated soybean meal	19.46	CP, %	22.73
Expanded soybean	11.00	SID CP, %	18.43
Low protein whey powder	10.00	SID Lys, %	1.43
Whey protein concentrate	4.00	SID Met + Cys, %	0.78
Fishmeal	3.00	SID Thr, %	0.84
Soybean oil	2.82	SID Trp, %	0.26
Sucrose	2.00	SID Ile, %	0.85
Soybean hulls	2.00	SID Val, %	0.91
Lysine HCl	0.33	SID Leu, %	1.64
DL-Methionine	0.14	SID Lys/ME (g/MJ)	5.49
L-Threonine	0.09	Ca, %	0.79
Calcium hydrophosphate	0.80	STTD P, %	0.62
NaCl	0.35	Na, %	0.31
Limestone powder	1.80		
Premix ^1^	1.00		
Total	100.00		

^1^ Supplied per kilogram of complete diet: Fe, 120 mg; Cu, 10 mg; Zn, 120 mg; Mn, 35 mg; I, 0.25 mg; Se, 0.2 mg; Vitamin A, 8000 IU; Vitamin D3, 1000 IU; Vitamin E, 30 mg; Vitamin K3, 2 mg, Vitamin B1, 2 mg; Vitamin B2, 6 mg; Vitamin B6, 4.0 mg; Vitamin B12, 0.02 mg; Niacin, 25 mg; Calcium pantothenate, 10 mg; Folic acid, 1.0 mg; Biotin, 0.25 mg. ^2^ Nutrient profiles were derived following the computational guidelines outlined in the NRC (2012) database.

**Table 2:** Primer sequences in the study.

Genes	Primer Sequence (5′-3′)	GenBank	Product Size (bp)
*β-actin*	F: TGCGGGACATCAAGGAGAAGC	XM_021086047	273
	R: ACAGCACCGTGTTGGCGTAGAG		
*MUC*	F: CTGCTCCGGGTCCTGTGGGA	XM_021082584.1	101
	R: CCCGCTGGCTGGTGCGATAC		
*pBD-1*	F: ACCGCCTCCTCCTTGTATTC	NM_213838.1	150
	R: CACAGGTGCCGATCTGTTTC		
*pBD-2*	F: CCAGAGGTCCGACCACTACA	NM_214442.2	88
	R: GGTCCCTTCAATCCTGTTGAA		
*PG1*	F: GTAGGTTCTGCGTCTGTGTCG	NM_001123149.2	166
	R: CAAATCCTTCACCGTCTACCA		

**Table 3:** Effects of GLU on growth performance and diarrhea rate in piglets.

Item	CON	GLU	SEM	*p*-Value
Initial BW, kg	6.33	6.32	0.13	0.979
Final BW, kg	12.97 ^a^	14.25 ^b^	0.36	0.02
ADG, g/d	315.95 ^a^	374.0 ^b^	19.63	0.038
ADFI, g/d	414.12 ^ab^	490.74 ^ab^	20.28	0.026
G/F	0.77	0.76	0.03	0.973
Diarrhea rate, %	24.45 ^a^	15.00 ^b^	2.58	0.035

CON, control group; GLU, glucuronolactone group; BW, body weight; ADG, average daily gain; ADFI, average daily feed intake; G/F, gain/feed ratio. The data are shown as the mean ± standard error (*n* = 6). Different letters (ab) indicate significant differences (*p* < 0.05).

## Data Availability

Please contact the author if further information is required.

## Supplementary Materials

The following supporting information can be downloaded at: https://www.mdpi.com/article/10.3390/antiox14030352/s1, The raw sequence data of the ileal microbiota reported in this paper have been deposited in the Genome Sequence Archive (Genomics, Proteomics & Bioinformatics) in National Genomics Data Center, China National Center for Bioinformation that are publicly accessible at https://ngdc.cncb.ac.cn/search/all?q=CRA017814 (accessed on 18 July 2024). Table S1: ELISA kit information in the study; Table S2: Antibody information used in the study.
