# Supplementary material for: Glucuronolactone Restores the Intestinal Barrier and Redox Balance Partly Through the Nrf2/Akt/FOXO1 Pathway to Alleviate Weaning Stress-Induced Intestinal Dysfunction in Piglets
Source: Antioxidants (Basel). 2025 Mar 18;14(3):352. doi: 10.3390/antiox14030352 (PMC11939252; doi:10.3390/antiox14030352)
Supplement: Supplementary file 1 [file antioxidants-14-00352-s001.zip › antioxidants-3489178-supplementary.pdf]

Supplementary Material

Table S1 Elisa kit information in the study

Items	Company	Product code
TP	Nanjing Jiancheng, China	A045-2-2
ALB	Nanjing Jiancheng, China	A028-2-1
TBili	Nanjing Jiancheng, China	C019-1-1
ALT	Nanjing Jiancheng, China	C009-2-1
AST	Nanjing Jiancheng, China	C010-2-1
ALP	Nanjing Jiancheng, China	A059-2-2
T-AOC	Nanjing Jiancheng, China	A015-2-1
T-SOD	Nanjing Jiancheng, China	A001-3-2
CAT	Nanjing Jiancheng, China	A007-1-1
MDA	Nanjing Jiancheng, China	A003-1-2
DAO	Jiangsu Meimian, China	MM-043801
D-lactate	Jiangsu Meimian, China	MM-3373202
TGF-β	Jiangsu Meimian, China	MM-042201
IL-6	Jiangsu Meimian, China	MM-041801
TNF-α	Jiangsu Meimian, China	MM-038301
IL-22	Jiangsu Meimian, China	MM-123101
IL-1β	Jiangsu Meimian, China	MM-042201
IL-10	Jiangsu Meimian, China	MM-042501
sIgA	Jiangsu Meimian, China	MM-3623401
IgG	Jiangsu Meimian, China	MM-040301
IgM	Jiangsu Meimian, China	MM-040201

Table S2 Antibody information used in the study

Name	Brand	Product code
Akt	Cell Signaling Technology, USA	9272S
p-Akt	Cell Signaling Technology, USA	4060S
FOXO1	Cell Signaling Technology, USA	2880S
SOD1	Cell Signaling Technology, USA	2770S
p38	Cell Signaling Technology, USA	9212S
p-p38	Cell Signaling Technology, USA	4511S
TLR4	Proteintech, USA	66350-1
TAB1	Cell Signaling Technology, USA	3226S
MyD88	Cell Signaling Technology, USA	4283S
Nrf2	LifeSpan, USA	A003-1-2
p-Nrf2	Cell Signaling Technology, USA	MM-0422O1
Keap1	Cell Signaling Technology, USA	48768T
NFκB	proteintech, USA	10745-1-AP
p-NFκB	Cell Signaling Technology, USA	3033S
ZO-1	Cell Signaling Technology, USA	13663S
Occludin	Abcam, USA	ab31721
Claudin-1	Abcam, USA	ab15098
β-actin	Beyotime, Shanghai, China	AF5003
